# Personality traits can predict which exercise intensities we enjoy most, and the magnitude of stress reduction experienced following a training program

**DOI:** 10.3389/fpsyg.2025.1587472

**Published:** 2025-07-08

**Authors:** Flaminia Ronca, Benjamin Tari, Cian Xu, Paul W. Burgess

**Affiliations:** ^1^Institute of Sport, Exercise and Health, University College London, London, United Kingdom; ^2^Institute of Cognitive Neuroscience, University College London, London, United Kingdom

**Keywords:** Big Five, physical activity, neuroticism, exercise tailoring, fitness

## Abstract

**Introduction:**

The aim of this study was to determine if personality can predict physical fitness, enjoyment of exercise by intensity, and engagement in an exercise program in the general population.

**Methods:**

Participants were assigned to either an 8-week home-based cycling and strength training intervention or a resting control group.

**Results:**

Personality traits were strong predictors of baseline fitness levels, and of enjoyment of different exercise intensities. For example, conscientiousness predicted greater general fitness and more weekly hours of physical activity, whereas extraversion and neuroticism predicted higher V̇O_2peak_ and poorer heart rate recovery, respectively. Extraversion also predicted greater enjoyment of highest intensity activities, whereas neuroticism predicted lower enjoyment of activities which required sustained effort. Importantly, those who scored high on neuroticism benefited the most from potential stress-reducing effects of aerobic training.

**Discussion:**

These findings provide insight into how personality can determine engagement with physical activity, and the degree to which one enjoys different forms of exercise, thus aiding the development of tailored exercise programs.

## Introduction

The World Health Organisation (WHO) physical activity recommendations indicate that healthy adults should complete at least 150 min of activity (i.e., strength, endurance, mixed) per week (Physical activity, n.d.); however, only 22.5% of adults and 19% of adolescents worldwide achieve these goals (Guthold et al., 2018). Accordingly, physical inactivity is becoming one of the leading risk factors for poor physical and mental health across the lifespan (Farooq et al., 2020), and there is a growing need for effective ways to encourage participation in physical activity. Understanding how individual personality traits relate to physical activity engagement may help strengthen the efficacy of such interventions and shape physical education practice in schools to promote positive affect and enjoyment during exercise (Reed and Buck, 2009; Posadzki et al., 2020).

Personality has been shown to impact health behavior uptake and thus the onset of disease and comorbidities. For example, neuroticism has been associated with maladaptive life choices and an increased prevalence of cardiovascular disease (Kim, 2022). Notably, when a person who scores high on neuroticism also exhibits high conscientiousness, they are more likely to engage in healthy behaviors (Graham et al., 2020). Similarly, although extraversion has been associated with smoking and heavy drinking, this trait is also associated with more physical activity; thus, these individuals tend to exhibit a higher incidence of cardiovascular disease but not metabolic syndrome (Kim, 2022). Therefore, understanding the effect of personality on physical activity behaviors can inform public health research and increase the effectiveness of physical activity-related interventions. There is a large body of literature outlining the effects of personality on performance in athletic populations (Egloff and Jan Gruhn, 1996; Allen et al., 2013) and additional studies on exercise adherence in clinical populations (Daley, 2002; Kruger et al., 2018).

The available literature that explores the above relationship has focused primarily on the Big Five personality traits (i.e., extraversion, conscientiousness, agreeableness, neuroticism, and openness) (McCrae and Costa, 1997) which are the most used in the field of sport and exercise psychology. Extraversion has been related to greater levels of physical activity (Rhodes and Pfaeffli, 2012) and greater engagement in organized sport (Engels et al., 2022); conscientiousness has been associated with greater commitment towards physical activity (Rhodes et al., 2005) and more active lifestyles (Conner and Abraham, 2001); agreeableness was shown to relate to positive experience in sport (Sutin et al., 2016); and neuroticism has been largely associated with a reduced willingness to exercise which is potentially related to engagement anxiety (Courneya and Hellsten, 1998; Engels et al., 2022). This anxiety sometimes leads to physical inactivity (Rhodes and Pfaeffli, 2012; Sutin et al., 2016). Apart from openness, the Big Five personality traits have been consistently associated with some aspect of engagement in physical activity, and this suggests that personality predicts one’s willingness and commitment to partake in regular physical activity.

The above associations have been largely assessed via cross-sectional self-reported questionnaires on physical activities which risk exposure to recall bias. Perceived levels of fitness and physical activity are notoriously difficult to measure using subjective questionnaires (Shephard, 2003). Although there are numerous cross-sectional studies relating personality to sporting success (Laborde et al., 2020), these typically focus on the performance of athletic populations and are not necessarily generalizable to the wider population. The relationships reported thus far call for further research to corroborate these findings through more objective measurements, immediate recall, and longitudinal designs to test the effects of exercise interventions on various personality types. Determining whether personality traits can predict engagement in exercise programs will help behavioral professionals target areas of need, design tailored sessions and interventions to promote positive affect and enjoyment with exercise, and ensure long term behavior changes (Reed and Buck, 2009; Posadzki et al., 2020).

Therefore, this study aimed to (i) identify relationships between personality and baseline fitness levels, (ii) determine whether personality influenced enjoyment of specific forms of exercise, and (iii) determine whether personality influenced the outcomes of a training intervention. Accordingly, we expect that individuals’ scores on extraversion, neuroticism, conscientiousness and agreeableness, but not openness, will be directly related to these outcomes.

## Methods

### Participants

Participants were recruited from the general public via email newsletters (i.e., company-and university-wide emails) and social media advertisements (i.e., via LinkedIn, Facebook, etc.). A total of 232 participants had expressed interest in participating in this study (i.e., university students: 33, emergency workers, police or health services: 134; other: 65), of which 132 attended the laboratory for pre-intervention testing. Participants were then match-randomised into an intervention and a control group by age, birth sex, BMI, and V̇O_2peak_. Of these, 86 completed the entire study protocol. Reasons cited by participants for leaving the study included illness (*n* = 2), surgery (*n* = 3), and/or general unavailability or loss of contact (*n* = 41). Participants were excluded from the study if they presented any physical illness or injury that prevented them from safely taking part in physical activity, determined via the physical activity readiness questionnaire (PAR-Q). The sample included here was determined adequate to detect statistically significant changes (mean change 5 ± 5, power 0.80, alpha = 0.05). All participants provided informed consent prior to being enrolled in the study and ethical approval was granted by the University College London Research Ethics Committee (13985/004). This study was conducted in line with guidelines presented in the Declaration of Helsinki. Data will be made available on request.

### Study measures

Prior to attending the laboratory sessions, participants completed an online questionnaire which included demographic information, the Perceived Stress Scale 10 items (PSS-10) (Cohen and Williamson, 1988), and a modified version of the Big Five Inventory 10 item (BFI-10) (Rammstedt and John, 2007). The former is a widely implemented, 10-item scale designed to assess individuals’ perceived stress. The latter is a 10-item scale used to assess personality traits (i.e., Extraversion, Agreeableness, Conscientiousness, Emotional Stability, and Openness) wherein each trait is represented by 2 questions. Here, we included a third Agreeableness item (“Is considerate and kind to almost everyone”) as recommended by Rammestedt and John to improve the measurement of this personality trait (Rammstedt and John, 2007). Factor analysis demonstrated that this 11^th^ item exhibited a loading of 0.89 onto the Agreeableness factor, confirming its appropriateness in the scale. The PSS-10 has been widely recognized for its reliable assessment of perceived stress and Cronbach’s alpha levels have been reported between 0.65 and 0.93 (Roberti et al., 2006). In contrast, studies employing the BFI-10 reported Cronbach’s alpha scores between 0.14 and 0.71 (Kwon and Park, 2016; Abdul Azis et al., 2024); however, BFI-10 has demonstrated acceptable test–retest reliability and correlates well with the BFI-44 (Rammstedt and John, 2007).

### Study design

**Figure 1:**
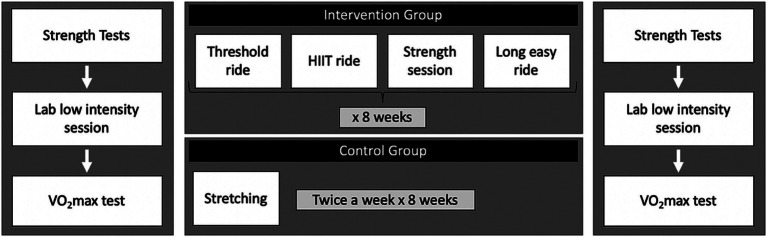
Study Design demonstrating the order in which exercise sessions were carried out. Participants rated how much they enjoyed their session after each lab task and after each home-based session in week 1 only.

Participants then attended the laboratory for baseline fitness testing (i.e., V̇O_2peak_) and were match-randomized to either an intervention or resting control group based on their age, gender, and baseline fitness level following the above-mentioned baseline assessments. The intervention group were provided with a home-based 8-week cycling and strength training plan, whereas participants assigned to the control condition were asked to maintain their normal lifestyle and were provided a plan of weekly 10-min stretching exercises. These were provided as a form of engagement for the control group, but these individuals were not monitored. During the laboratory tests, and during week 1 of the training period, participants were asked to rate their enjoyment of each training session from 1 to 7 (i.e., 1 being not enjoyable and 7 being extremely enjoyable). After the 8-week intervention, all participants completed the PSS-10 questionnaire a second time and attended the laboratory for post-intervention testing (Figure 1).

### Laboratory testing

Prior to maximal exercise testing, body composition was estimated using bioelectrical impedance on a Tanita MC980MA (Tanita Corporation, Tokyo, Japan). Participants then undertook three strength tests completed on the same day, separated by 4 min of rest between each test. These tests included: press-ups (i.e., maximum number of press-ups on hands and toes, not knees, in 1 min); countermovement jumps with hands on hips on a force plate (Hawkin Dynamics Inc., Maine, USA); and plank (i.e., on elbows and forearms and toes) to failure. Following strength exercise testing, participants completed the ‘Lab low intensity session’, rested for 30 min, and then completed the V̇O_2peak_ test. The Lab low intensity session was only completed once at pre-or post-intervention testing and when participants completed this session was determined via random order generator. Participants rated their session enjoyment (i.e., 1–7 as above) immediately after the low intensity session, and immediately after the V̇O_2peak_ test.

### Lab low intensity session

The exercise session required participants to complete 15 min of low-intensity cycling (i.e., rating of perceived exertion (RPE) of 4) on a stationary cycle ergometer (Corvial OEM, Lode BV Medical Technology, Groningen, The Netherlands), while heart rate (Polar H9, Polar, Finland) and breath-by-breath gas exchange (Vyntus CPX Metabolic Cart, Vyaire Medical Inc., USA) were monitored, respectively. The researchers controlled the required exercise intensity by adjusting the load on the ergometer to maintain a constant RPE of 4 out of 10 using the visual OMNI scale (Robertson and Noble, 1997). Intensity was adjusted as needed for each participant.

### V̇O_2peak_ test

The V̇O_2peak_ test was conducted using the same cycle ergometer and physiological monitoring equipment as the low intensity session. Participants completed 3 min of loadless cycling prior to the onset of a ramp protocol which continued for the remainder of the test. The workload was increased by either 15 or 25 W/min depending on the participant’s self-reported training level. Participants were instructed to maintain a constant cadence of between 60 and 70 rpm during testing. Verbal encouragement by the researchers was not provided in order to obtain accurate assessments of participants’ own motivation to exercise. Participants were instead updated on the test time every 2 min with a neutral tone of voice. Researchers did not otherwise engage with participants during testing. The V̇O_2peak_ test was terminated upon volitional exhaustion, or if the participant’s cadence dropped below 60 rpm for 30 s, or twice within a 45 s period. Participants completed a recovery phase pedaling for 3 min at a workload of 25 W. Heart rate recovery (HRR) was monitored as the change in heart rate from the time of reaching its maximum value to 120 s into recovery. V̇O_2peak_ and peak respiratory exchange ratios (RER_peak_) were determined following data smoothing via averaging every 7-breaths as the highest values obtained during testing, and the anaerobic threshold was determined using the v-slope method, respectively (Beaver et al., 1986).

### Exercise intervention sessions

Participants were provided with a Polar H9 (Polar, Finland) chest strap to monitor their heart rate during exercise sessions across the 8-week intervention via the Polar Beat app 3.5.0 (Polar, Finland). Exercise-related thresholds were used to determine five heart rate zones which were individual to the participant using Polar Flow (Polar, Finland) on participants’ phones. To determine the zones, heart rate (HR) values corresponding to the aerobic threshold (VT1), anaerobic threshold (VT2), and peak HR were extracted from the V̇O_2peak_ test and inputted into the Polar Beat application, which utilized these values as reference points to delineate the five participant-specific zones. Specifically, lower-intensity zones were determined relative to the aerobic threshold, moderate-intensity zones were positioned between the aerobic and anaerobic thresholds, and high-intensity zones were determined in relation to the anaerobic threshold and peak HR.

The intervention consisted of a cycling program which included three endurance sessions (Easy long, Threshold, High intensity interval training (HIIT)) as well as one strength session per week. During the first week of the training program participants were asked to log their session enjoyment during each session. The ‘Easy long ride’ consisted of 50 min of cycling at an intensity inducing a heart rate within zone 2; the ‘Threshold ride’ consisted of cycling at variable intensities over the course of the ride period and included 15 min within zone 2, 5 min within zones 3–4, and 5 min within zone 2; last, the ‘HIIT ride’ consisted of cycling for 10 min at an intensity where heart rate would fall within zone 2 as well as 4, 2 min rides completed at maximal effort with 2 min of active recovery (i.e., slow cadence pedaling), and 5 min of cycling at an intensity to achieve a heart rate in zone 2. The intensity of each session gradually increased over the course of the 8-week intervention. Finally, the ‘Strength session’ consisted of body weight exercises including squats, lunges, press-ups, sit-ups, calf raises, and glute bridges completed for 3 sets with 8 repetitions in each set. Progression and regression versions of each exercise were provided where participants were instructed to adjust the level of difficulty to target an RPE of 8 over the course of the 8-week intervention. Participants were asked to continue to log their perceived enjoyment after each aerobic and strength session during this time.

### Data analysis

All statistical analyses were conducted in RStudio (Posit | The Open-Source Data Science Company, n.d.). Data were checked for normality using the Shapiro–Wilk test. As the personality trait distribution was not normally distributed, non-parametric analyses were used. Demographic comparisons between groups were checked through independent t-tests or Mann Whitney U tests. Pre-post intervention changes were assessed using a mixed model ANOVA and multiple linear regressions (backward elimination) were implemented to examine relationships between personality traits and physical variables or enjoyment. Logistic regressions by backward elimination were implemented in the same manner to predict adherence likelihoods. All five personality traits, age, and gender were included in the full models where variables with the lowest AIC were removed in turn until all variables met *p* < 0.05. The *α* level was set to *p* < 0.05 in all tests.

## Results

### Demographic overview

One hundred and thirty-two participants (56 female) attended baseline fitness testing in the laboratory (Table 1) and were match-randomized to intervention (*n* = 78) and control (*n* = 54) groups by age, birth sex, BMI, and V̇O_2peak_, such that these values did not differ between groups at baseline testing. Of these, 86 participants completed both pre-and post-testing for the control (*n* = 25) and intervention (*n* = 51) groups. Drop-outs were due to injury, illness, and loss of contact.

**Table 1:** Mean ± SD for main baseline physical fitness indicators and personality traits.

	Score ≥1	All (*n* = 132)	Female (*n* = 56)	Male (*n* = 76)
Age		38 ± 13	35 ± 13	39 ± 12
Fat %		21.9 ± 6.6	25.9 ± 5.6 ***	19.3 ± 5.9
V̇O_2peak_ (ml/min/kg)		38.8 ± 9.6	35.4 ± 8.7	41.3 ± 9.5 ***
Power output (w)		255 ± 77	199 ± 58	295 ± 62 ***
Press-ups (n/60s)		23 ± 17	11 ± 10	31 ± 15***
Extraversion	58%	0.9 ± 2.0	1.4 ± 2.0 *	0.6 ± 2.0
Conscientiousness	74%	1.7 ± 1.9	2.1 ± 1.8	1.5 + 1.9
Agreeableness	69%	1.4 ± 1.5	2.0 ± 1.3 ***	0.9 ± 1.4
Neuroticism	29%	−0.6 ± 2.0	0 ± 1.9 **	−1.1 ± 1.9
Openness	77%	1.6 ± 1.6	2.0 ± 1.6 *	1.4 ± 1.6

Significant differences between females and males are denoted as *** *p* ≤ 0.001, ** *p* ≤ 0.01, * *p* ≤ 0.05.

Females had a higher body fat percentage (*p* < 0.001), lower V̇O_2peak_ (*p* < 0.001), lower peak power output (*p* < 0.001), and completed fewer press-ups in 1 min than males (*p* < 0.001). In terms of personality traits, females scored higher than men on ratings of extraversion (*p* = 0.035), agreeableness (*p* < 0.001), neuroticism (*p* = 0.002) and openness (*p* = 0.041). Age was positively correlated with conscientiousness (*p* = 0.001) and negatively correlated with neuroticism (*p* < 0.001) (Table 2).

**Table 2:** Means, standard deviations and spearman correlations between personality traits and age (*n* = 132).

	*M*	SD	1	2	3	4	5	6
1. Age	37.65	13.09						
2. Sex	NA	NA	0.15					
3. Extraversion	0.92	2.04	0.16	−0.19*				
4. Neuroticism	−0.61	1.99	−0.31***	−0.28**	−0.20*			
5. Conscientiousness	1.73	1.88	0.28**	−0.16	0.22**	−0.25**		
6. Agreeableness	1.37	1.45	−0.01	−0.33***	0.08	−0.09	0.10	
7. Openness	1.61	1.62	0.06	−0.16	0.33***	−0.20*	0.23**	0.21*

Point biserial correlations for sex (Female = 0, Male = 1). * *p* ≤ 0.05, ** *p* ≤ 0.01, *** *p* ≤ 0.001.

As for study groups, participants in the control and intervention group did not differ according to their ratings of extraversion (control 0.8 ± 2.1, intervention 1.0 ± 2.0), neuroticism (control −0.3 ± 2.0, intervention −0.8 ± 1.9), conscientiousness (control 1.6 ± 1.7, intervention 1.8 ± 2.9), agreeableness (control 1.4 ± 1.3, intervention 1.3 ± 1.5) nor openness (control 1.5 ± 1.5, intervention 1.7 ± 1.7) (*p*s > 0.16).

### Pre-intervention measurements

**Figure 2:**
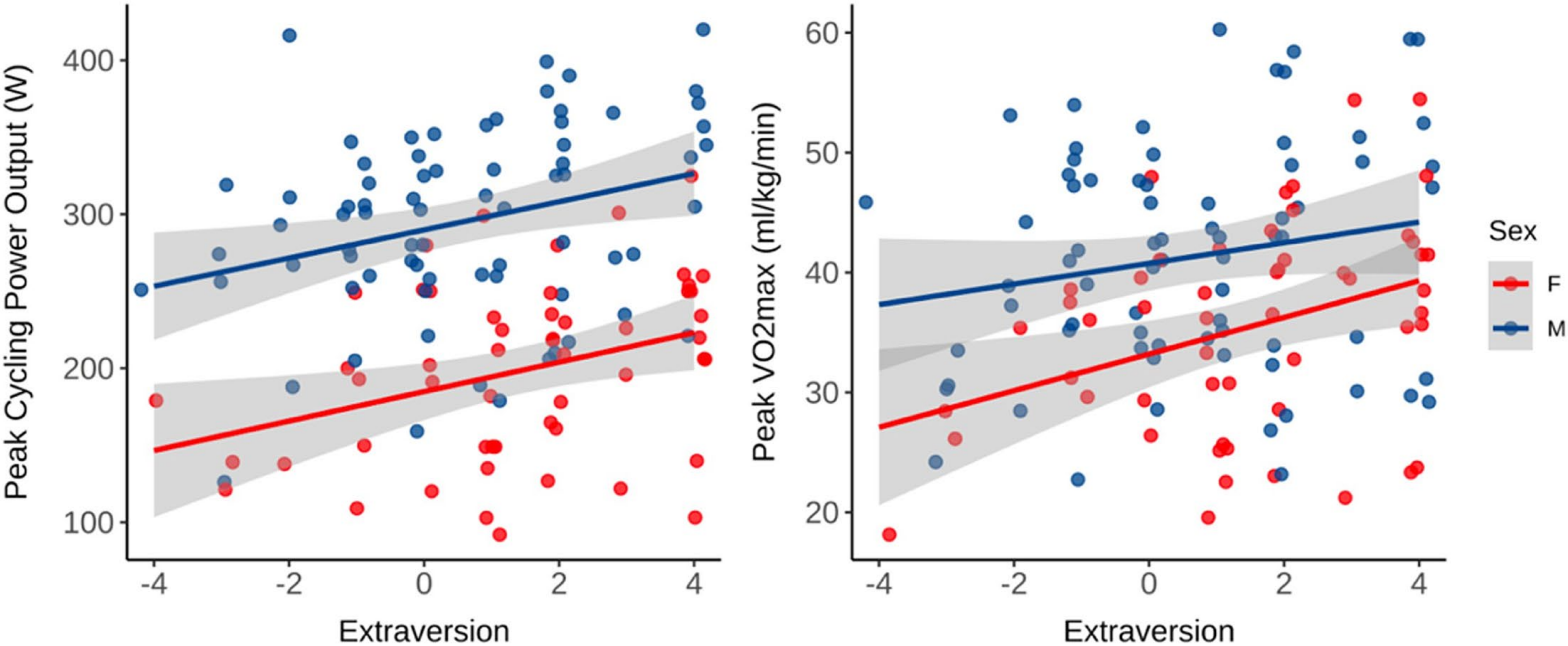
Regressions of extraversion on V̇O_2peak_ (*R*2 = 0.15, *p* < 0.001) and Peak cycling power output (*R*2 = 0.16, *p* < 0.001) on a cycling V̇O_2peak_ test. Birth sex (*n* = 132, female = 56) was also a significant predictor in both models (*p* < 0.001), see Table 3.

**Figure 3:**
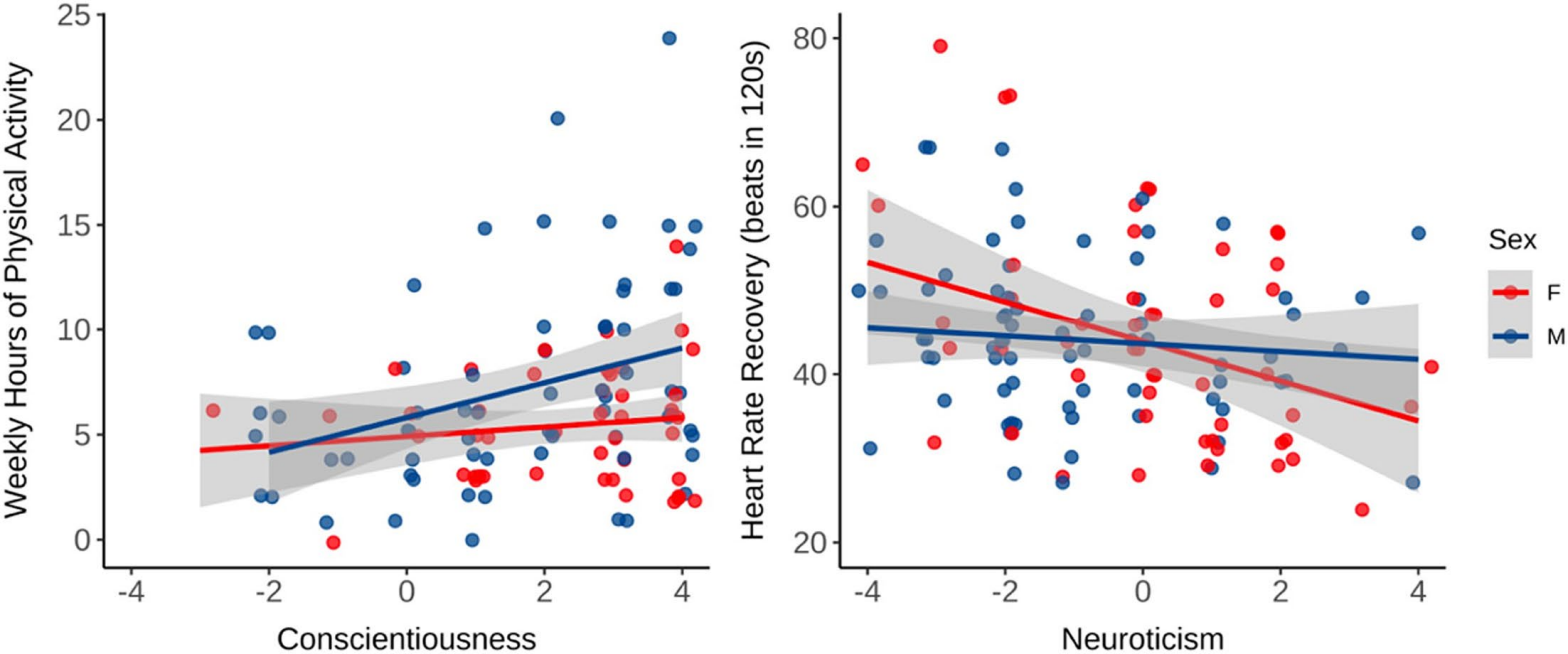
Regressions of conscientiousness on self-reported weekly hours of physical activity (*R*2 = 0.12, *p* = 0.001), and of neuroticism on HRR after a V̇O_2peak_ test (R2 = 0.13, *p* = 0.003). Gender was a significant predictor in the conscientiousness model (*p* = 0.014) but not in the neuroticism model; (*n* = 132, female = 56), see Table 3.

To determine if personality traits predicted fitness measures, stepwise multiple linear regressions using backward elimination were conducted for each variable. All five traits, age, and gender were included in the full model (Table 3). Of the five personality traits, only extraversion was predictive of having higher V̇O_2peak_, anaerobic threshold, and peak power output, *Fs*(2,123) > 11.96, *p*s < 0.001, *R*^2^_adj_ > 0.13, respectively (Figure 2). Conscientiousness predicted press-up completion, longer plank times, more weekly hours of physical activity, and lower body fat percentage, *Fs*(3,121) > 4.03, *p*s < 0.046, *R*^2^_adj_ > 0.02, respectively. Neuroticism only predicted poorer HRR, *F*(1,69) = 9.98, *p* = 0.002, *R*^2^_adj_ = 0.11 (Figure 3). Openness and agreeableness were not predictive in any model for baseline fitness variables. Muscle mass was only predicted by age and sex, *Fs*(2,125) > 128.8, *p*s < 0.001, *R*^2^_adj_ = 0.67.

**Table 3:** Multiple linear regression output for each component of baseline physical fitness following backward elimination.

	Estimate	95% CI [LL, UL]	*p*	df	*F*	*R* ^2^	*R* ^2^_adj_
DV: V̇O_2peak_					
Extraversion	1.15	[0.36, 1.93]	0.006				
Sex (male)	6.83	[3.60, 10.10]	<0.001				
Regression			<0.001	2, 120	13.38	0.15	0.13
DV: anaerobic threshold					
Extraversion	1.40	[0.74, 2.05]	<0.001				
Sex (male)	4.57	[1.84, 7.30]	0.002				
Regression			<0.001	2, 120	11.33	0.16	0.14
DV: peak power output					
Extraversion	8.06	[3.01, 13.11]	0.003				
Age	0.98	[0.18, 1.78]	0.018				
Sex (male)	99.56	[79.70, 120.40]	<0.001				
Regression			<0.001	3, 119	35.4	0.47	0.46
DV: heart rate recovery					
Neuroticism	−2.19	[−3.58, −0.81]	0.003	1, 66	9.54	0.13	0.12
DV: press-ups					
Conscientiousness	2.17	[0.91, 3.36]	<0.001				
Age	−0.29	[−0.47, −0.11]	0.001				
Sex (male)	23.32	[18.74, 27.90]	<0.001				
Regression			<0.001	3, 118	33.13	0.48	0.44
DV: plank time					
Conscientiousness	3.97	[0.06, 7.89]	0.046	1, 122	4.06	0.03	0.02
DV: PA weekly hours					
Conscientiousness	0.62	[0.22, 1.22]	0.002				
Sex (male)	2.15	[0.64, 3.66]	0.014				
Regression			0.001	2, 106	7.20	0.12	0.11
DV: body fat %					
Conscientiousness	−0.56	[−1.10, −0.01]	0.039				
Age	0.20	[.12, 0.28]	<0.001				
Sex (male)	−7.76	[−9.80, −5.62]	<0.001				
Regression			<0.001	3, 124	23.43	0.36	0.35
DV: muscle mass					
Age	0.18	[0.09, 0.27]	<0.001				
Sex (male)	17.96	[15.61, 20.30]	<0.001				
Regression			<0.001	2, 125	128.8	0.67	0.67

The full models included all Big Five personality traits, age and sex. Full models included all Big Five personality traits + Sex + Age, predictors were removed via backward elimination until all variables were significant (*p* < 0.05).

Participants who were part of an endurance club (*n* = 45) were more extraverted (*p* = 0.039) and more conscientious (*p* = 0.007) than those who were not. However, following backward elimination during logistic regression, only conscientiousness remained as a significant predictor of this factor (OR: 1.28, *p* = 0.021).

### Exercise enjoyment

Participants who scored higher in neuroticism reported less enjoyment of the low intensity laboratory session and the threshold ride, *F*s(1,53) > 4.27, *p*s < 0.009, *R*^2^_adj_ = 0.15. In contrast, higher extraversion predicted enjoyment of the V̇O_2peak_ test and the HIIT sessions, *Fs*(1,125) > 5.10, *p*s < 0.026, *R*^2^_adj_ > 0.03, respectively, whereas openness predicted less enjoyment of the threshold ride and the HIIT rides. Agreeableness predicted more enjoyment of the easy long ride, *F*(2,49) = 6.08, *p* = 0.004, *R*^2^_adj_ = 0.17 (see Table 4).

**Table 4:** Multiple linear regression outputs predicting enjoyment of each exercise session with personality traits following backward elimination.

	Estimate	CI [LL, UL]	*p*	df	*F*	*R* ^2^	*R* ^2^_adj_
DV: enjoyed stretching					
Neuroticism	0.18	[0.03, 0.33]	0.023	1, 32	5.58	0.15	0.12
DV: enjoyed strength session					
Sex (male)	0.10	[−0.04, 0.24]	0.18	1, 50	1.99	0.04	0.03
DV: enjoyed lab low intensity session					
Neuroticism	−0.25	[−0.42, −0.08]	0.005	1, 43	8.74	0.17	0.15
DV: enjoyed easy long ride					
Agreeableness	0.24	[0.01, 0.27]	0.049	1, 42	4.11	0.09	0.07
DV: enjoyed threshold ride					
Neuroticism	−0.19	[−0.35, −0.03]	0.024	1	1.29		
Openness	−0.25	[−0.40, −0.09]	0.005	1	5.13		
Sex (male)	−0.63	[−1.21, −0.05]	0.031	1	4.73		
Regression			0.015	3, 51	3.85	0.18	0.14
DV: enjoyed high intensity interval ride					
Extraversion	0.21	[0.01, 0.43]	0.031	1	0.68		
Openness	−0.40	[−0.65, −0.15]	0.004	1	9.10		
Regression			0.012	1, 46	4.89	0.18	0.14
DV: enjoyed lab V̇O_2peak_ test					
Extraversion	0.13	[0.02, 0.25]	0.039	1, 117	4.32	0.04	0.03

The full models included all Big Five personality traits, age and sex.

### Personality effects on program adherence and participation

Participants in the intervention group who scored higher on neuroticism were less likely to record their HR data required for research monitoring throughout the 8 weeks of training (OR: 0.73). This was independent of whether participants attended the laboratory for post intervention testing. Extraverted participants were less likely to attend post-intervention testing (OR: 0.70), whereas openness predicted a greater likelihood to attend (OR: 1.42).

### Intervention outcomes

Our results demonstrated group by time interactions for V̇O_2peak_, peak cycling power, total press-ups, and plank time, *Fs*(1,87) > 5.7, *p*s < 0.02, η_p_^2^ > 0.06, where only the intervention group exhibited pre-post improvements in these measures (*p*s < 0.001). No changes were observed for RER_peak_ and BMI (Table 5). Within the sample, and regardless of personality type, we observed a significant increase in weekly hours of exercise, V̇O_2peak_, peak power output, number of press-ups, and plank duration in the intervention group (*p*s < 0.02).

**Table 5:** Comparison of pre- and post-test changes in physical variables for the Intervention and Control groups.

	Control (*n* = 35)		Intervention (*n* = 51)	
	Pre	Post	Pre	Post
V̇O_2peak_ (ml/kg/min)	31.9 ± 11.6	40.2 ± 12.0	38.7 ± 8.40	41.4 ± 7.81***
Peak power output (W)	241 ± 92	243 ± 109	265 ± 66	280 ± 56***,†
RER_peak_	1.19 ± 0.09	1.21 ± 0.09	1.18 ± 0.07	1.17 ± 0.08
Press ups (n)	26 ± 18	23 ± 18	24 ± 12	32 ± 14***
Plank time (s)	125 ± 53	125 ± 57	136 ± 59	170 ± 73***,†††
BMI	23.2 ± 3.5	22.9 ± 3.4	25.1 ± 4.1	25.1 ± 4.0
Change in PA hours (self-reported rating −2 to 2)		0.0 ± 0.9		0.5 ± 0.9 *

Significantly higher than Pre-value within group *p < 0.05, ***p < 0.001. Significantly higher than control group within timepoint †*p* < 0.05, †††*p* < 0.001.

**Figure 4:**
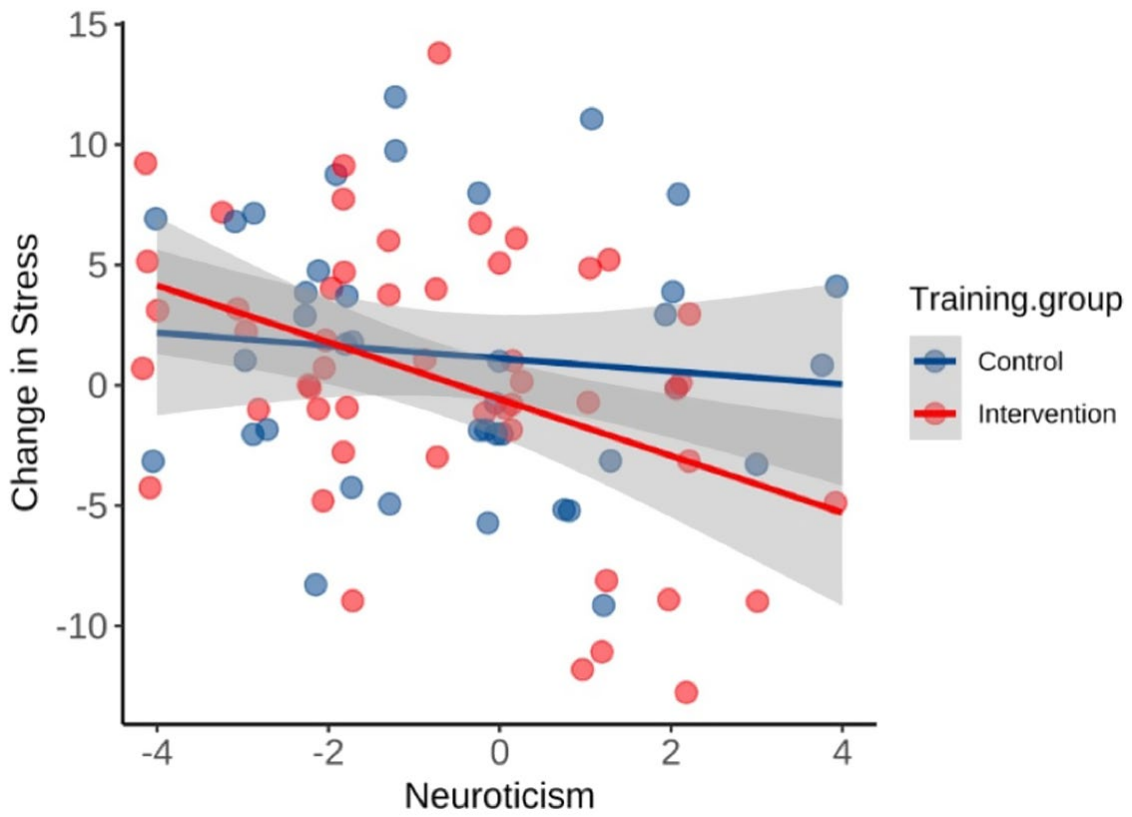
Relationship between neuroticism scores and changes in stress after the 8-week period. The prediction was significant in the intervention group only (*R*^2^ = 0.17, *p* = 0.003).

Multiple linear regression models were used to predict intervention outcomes by personality traits on the intervention group and the relationships between personality and program adherence are also explored (see Supplementary material). Here, more conscientious participants exhibited smaller improvements in peak power output over the course of the intervention, *F*(1,49) = 4.89, *p* = 0.032, *R*^2^_adj_ = 0.07; however, they reported that they had been asked to exercise fewer hours per week during the intervention compared to their usual weekly hours (although this effect trended toward significance; *p* = 0.06). Extraversion predicted an increase in RER_peak_ on the second laboratory visit, *F*(1,48) = 6.70, *p* = 0.013, *R*^2^_adj_ = 0.10. Furthermore, participants who scored high on neuroticism reported a greater decrease in stress after the intervention, *F*(1,49) = 9.94, *p* = 0.003, *R*^2^_adj_ = 0.15 (Figure 4). When adjusting for low, medium or high levels of neuroticism in a mixed model ANOVA, neuroticism was related to self-reported stress, *F*(2, 127) = 21.7, *p* < 0.001, and there was a significant intervention group by neuroticism level interaction, *F*(2,127) = 3.59, *p* = 0.031. In decomposing this interaction, perceived stress differed between low and the high neuroticism groups (*t* = 2.56, *p* = 0.012). Finally, a multiple linear regression by backward elimination revealed that post-intervention stress was predicted by the level of baseline stress and baseline V̇O_2peak_ (*t*s > −2.15, *p* = <0.035), but not post-intervention V̇O_2peak_.

## Discussion

This study aimed to determine whether personality traits were associated with physical fitness, and whether they could predict engagement with a home-based exercise program and its outcomes. Below we outline key findings and how they related to personality scores.

### Main study outcomes

#### Baseline fitness

Extraversion and conscientiousness predicted baseline fitness outcomes, but neuroticism did not. Instead, neuroticism did predict a poorer HRR following laboratory testing, independent of fitness. Such findings are consistent with neurotic sub-facets of anxiety and rumination (Watson and Casillas, 2003). In line with the literature, openness and agreeableness were not predictive of any baseline fitness measurements.

#### Session enjoyment and program adherence

Participants scoring high on extraversion enjoyed high intensity sessions the most and predicted a lower likelihood that participants would return for post-intervention testing. Individuals scoring high on neuroticism enjoyed light exercise sessions where they were not being watched (i.e., at-home light intensity sessions as opposed to the ‘lab low intensity’ cycling session), or sessions that did not require a lengthy sustained vigorous effort. Neuroticism did not predict dropout rates, but did predict a lower likelihood of self-monitoring heart rate on the online research platform. Those scoring high on openness rated strenuous exercise lower than other activities and this group was more likely to return for post intervention testing. Conscientiousness and agreeableness did not predict strong preferences of either intensity, and conscientiousness did not predict any of the adherence variables.

#### Intervention outcomes

The most notable outcome from the intervention was decreased stress only in those participants who scored high in neuroticism. This effect was predicted by higher baseline V̇O_2peak_ but was not related to improvements in fitness. In addition, conscientiousness did not predict greater improvements in peak cycling power output, and extraversion did not predict greater fitness improvements following the program. Therefore, it may be precarious to assume that personality traits influence the *magnitude* of fitness benefits gained from a home-based exercise program. However, it does appear that exercise programs may benefit specific personalities in different ways, as discussed in more detail below. For example, participants who scored highly on extraversion have been shown to be more likely to engage in sport (Engels et al., 2022), and performance is further predicted by higher scores of extraversion in athletic populations (McAuley et al., 2022). According to Eysenck’s (1976) biological theory of arousal, extraverts possess a lower resting state of arousal than introverts and therefore seek greater stimulation (Stelmack, 1990). This biological basis of extraversion underpins most of the findings reported in this study. That is, extraversion was predictive of greater enjoyment only of the V̇O_2peak_ and HIIT sessions (i.e., the highest exercise intensities). The mechanisms which explain greater engagement of extraverts are also largely related to the findings pertaining to neuroticism in this study.

Neuroticism has been associated with low engagement in physical activity (Rhodes and Smith, 2006) due to a higher association with perceived stress (Dunker et al., 2020) and greater focus on the fear of failure (Courneya and Hellsten, 1998). However, once other extrinsic factors are considered, this relationship appears to be weak (Rhodes and Pfaeffli, 2012). Graham et al. (2020) highlight the effects of “healthy neuroticism,” defined as an interaction between high neuroticism and high conscientiousness, and its significant association with greater engagement in physical activity. Therefore, the relationship between the broader trait of neuroticism and sport participation is complex and may require greater scrutiny of how sub-facets of the trait, and their interactions with other traits, predict attitudes towards specific forms of physical activity. The existing literature does indicate that neuroticism is associated with poorer cardiovascular recovery and highlights its deleterious effects on cardiac health in this population (Chida and Hamer, 2008). The Objective Self Awareness Theory (Duval and Wicklund, 1972) highlights the effects of an audience on increased introspection, prompting a comparison of the real self to the ideal self. Considering the higher emotional instability associated with neuroticism, it is possible that HIIT may be more tolerable than a long continuous vigorous session, where anxiety, worry and negative self-talk are more likely to impact performance (Altamura et al., 2019). In fact, enjoyment of physical activity has been associated with feelings of perceived competence in individuals with high neuroticism, who may prefer tasks that facilitate self-efficacy (Engels et al., 2022).

When considering conscientiousness, individuals who scored highly on this trait are more likely to be strongly motivated by health-protective behaviors (Conner and Abraham, 2001) and are more likely to translate intention to behavior (Rhodes et al., 2005). In exercise contexts, participants with high reward-dependence were found to be more likely to complete a 6-month training program (De Panfilis et al., 2008), and it is therefore unsurprising that conscientiousness was the only trait that did not predict enjoyment of any particular session on the program. This group might engage in physical activity largely for health purposes, where enjoyment might play a smaller motivational role compared to the reward of achieving their intended health or performance goals (Rhodes et al., 2005).

Openness is largely associated with intellect and curiosity and is characterized by high degrees of reflection and introspection (Connelly et al., 2014). Although this trait is sometimes related to adopting problem-solving coping mechanisms and to greater commitment in sporting contexts (Allen et al., 2013), it is generally not considered relevant to exercise engagement or performance. In a meta-analysis of 33 studies, Rhodes and Smith (2006) found that openness was not associated with any measure of engagement in physical activity, and according to Engels et al. (2022), openness is the only trait that does not relate to enjoyment of exercise. Therefore, the strong negative predictions of openness on enjoyment of higher intensity activities in this study are a new finding that somewhat contradicts current literature. It should be noted that the existing studies were based on recall questionnaires only, and did not measure the effects of exercise intensities on exercise enjoyment, as is reported here. It is worth noting, however, that openness has been associated with greater body awareness (Ferentzi et al., 2017), perhaps indicating that open-minded individuals may have a stronger tendency to focus on their sensations, leading to a higher likelihood of perceiving high levels of exertion as threatening if combined with neurotic traits.

Finally, in terms of agreeableness, two meta-analyses totaling 27 samples independently concluded that, once adjusting for other personality traits, there is no relationship between agreeableness and physical activity or sedentary behaviors (Rhodes and Smith, 2006; Sutin et al., 2016). This study supports these findings.

### Strengths and limitations

This study adds to literature on the direct relationship between personality, fitness, and exercise engagement in a healthy population, and provides good ecological validity for the reported associations. However, conducting lab-based tests with an interventional element did generate bias in the sample, where more than 70% of those who volunteered for the study were found to be open-minded, conscientious and emotionally stable. Sport participation history was not considered in this study. In line with the developmental hypothesis, potential changes in personality might arise through participation in sport determined by one’s upbringing or life events (Rhodes et al., 2005). Understanding the cyclical relationship between phenotypic and genotypic traits in exercise could provide a bases for physical activity promotion throughout childhood. Moreover, the present study sample were not explicitly asked about their motivation to participate in exercise. Rather, this was only ascertained via their performance during the study. The inclusion of this parameter would provide a more complete understanding of why participants performed the way they did. Last, the Big Five personality traits were implemented in this study without consideration of sub-traits or other personality factors, such as grit or anxiety. This may be relevant in the context of physical activity and is worth future research. The literature does not currently present an ‘exercise personality’ questionnaire which further constrains current research to rely on broader and more generalizable trait analyses. Nonetheless, this paper indicates that specific traits can strongly predict physical activity behaviors. Accordingly, the development of personality psychometrics that are more relevant to exercise engagement may prove useful in providing tailored recommendations for individuals.

### Implications and future directions

These results highlight that, although fitness was improved across personality types–for those who did complete the program - differences in enjoyment and adherence by personality traits suggest that tailoring exercise programs according to personality could potentially maximize these benefits. For example, the fact that extraversion predicted higher baseline scores on peak power output and V̇O_2peak_, greater enjoyment of high intensity home sessions, and greater effort to exhaustion in post-intervention lab tests (RER_peak_) suggests that extraverted individuals might particularly welcome the inclusion of high intensity aerobic sessions in a program. In contrast, while participants who scored high on neuroticism were less likely to monitor their heart rate during their sessions, they were just as likely to complete the program and return to the lab for post-intervention testing. These individuals exhibited a particularly strong reduction in stress following the exercise program. This suggests that individuals in this group might appreciate being given space for independence and privacy during an exercise program. Further studies could investigate if training plans that facilitate autonomy might be more welcomed by those who score highly on neuroticism, therefore supporting greater adherence for those less likely to engage in physical activity. Therefore, these results demonstrate a strong potential for the development of tailored programs according to personality traits. Research which identifies the optimal exercise-personality pairing should therefore be explored to support the development of effective exercise adherence strategies for behavior change, particularly in less active groups.

## Conclusion

In this study sample of members of the general public, the Big Five personality traits were strong predictors of existing physical activity behaviors, baseline fitness levels, and enjoyment of the differing prescribed exercise intensities. Group level improvements in fitness were observed regardless of personality profiles. Of note, neuroticism specifically predicted a significant reduction in self-reported stress, providing an encouraging outlook on the individualized stress-reducing effects of physical exercise. Overall, results provide insights into how personality traits influence exercise related behaviors, exercise enjoyment, and its long-term effects. These results demonstrate the potential utility of monitoring personality traits in future exercise studies and might aide the design of training programs tailored to participant’s needs.

## Data Availability

The raw data supporting the conclusions of this article will be made available by the authors, without undue reservation.
