# Correction: Personality traits can predict which exercise intensities we enjoy most, and the magnitude of stress reduction experienced following a training program

**DOI:** 10.3389/fpsyg.2025.1663653

**Published:** 2025-08-08

**Authors:** Flaminia Ronca, Benjamin Tari, Cian Xu, Paul W. Burgess

**Affiliations:** ^1^Institute of Sport, Exercise and Health, University College London, London, United Kingdom; ^2^Institute of Cognitive Neuroscience, University College London, London, United Kingdom

**Keywords:** Big Five, physical activity, neuroticism, exercise tailoring, fitness

In the published article, there was an error in the author list.

Author Benjamin Tari was erroneously assigned as corresponding author. The correct corresponding author is Flaminia Ronca.

There was a mistake in Tables 3, 4 as published. Both tables are missing the columns for estimates and confidence intervals. There was a mistake in the captions of Tables 3, 4 as published. A clarification has been added. The corrected Tables 3, 4, along with their updated captions appear below.

**Table 3:** Multiple linear regression output for each component of baseline physical fitness following backward elimination.

	**Estimate**	**95% CI [LL, UL]**	***p***	**df**	***F***	***R* ^2^**	** $R_{\text{adj}}^{2}$ **
**DV: VO**_2peak_							
Extraversion	1.15	[0.36, 1.93]	0.006				
Sex (male)	6.83	[3.60, 10.10]	< 0.001				
Regression			< 0.001	2, 120	13.38	0.15	0.13
**DV: anaerobic threshold**							
Extraversion	1.40	[0.74, 2.05]	< 0.001				
Sex (male)	4.57	[1.84, 7.30]	0.002				
Regression			< 0.001	2, 120	11.33	0.16	0.14
**DV: peak power output**							
Extraversion	8.06	[3.01, 13.11]	0.003				
Age	0.98	[0.18, 1.78]	0.018				
Sex (male)	99.56	[79.70, 120.40]	< 0.001				
Regression			< 0.001	3, 119	35.4	0.47	0.46
**DV: heart rate recovery**							
Neuroticism	−2.19	[−3.58, −0.81]	0.003	1, 66	9.54	0.13	0.12
**DV: press-ups**							
Conscientiousness	2.17	[0.91, 3.36]	< 0.001				
Age	−0.29	[−0.47, −0.11]	0.001				
Sex (male)	23.32	[18.74, 27.90]	< 0.001				
Regression			< 0.001	3, 118	33.13	0.48	0.44
**DV: plank time**							
Conscientiousness	3.97	[0.06, 7.89]	0.046	1, 122	4.06	0.03	0.02
**DV: PA weekly hours**							
Conscientiousness	0.62	[0.22, 1.22]	0.002				
Sex (male)	2.15	[0.64, 3.66]	0.014				
Regression			0.001	2, 106	7.20	0.12	0.11
**DV: body fat %**							
Conscientiousness	−0.56	[−1.10, −0.01]	0.039				
Age	0.20	[.12, 0.28]	< 0.001				
Sex (male)	−7.76	[−9.80, −5.62]	< 0.001				
Regression			< 0.001	3, 124	23.43	0.36	0.35
**DV: muscle mass**							
Age	0.18	[.09, 0.27]	< 0.001				
Sex (male)	17.96	[15.61, 20.30]	< 0.001				
Regression			< 0.001	2, 125	128.8	0.67	0.67

The full models included all Big Five personality traits, age and sex.

Full models included all Big Five personality traits + Sex + Age, predictors were removed via backward elimination until all variables were significant (*p* < 0.05).

**Table 4:** Multiple linear regression outputs predicting enjoyment of each exercise session with personality traits following backward elimination.

	**Estimate**	**CI [LL, UL]**	***p***	**df**	***F***	***R* ^2^**	** $R_{\text{adj}}^{2}$ **
**DV: enjoyed stretching**							
Neuroticism	0.18	[0.03, 0.33]	0.023	1, 32	5.58	0.15	0.12
**DV: enjoyed strength session**							
Sex (male)	0.10	[−0.04, 0.24]	0.18	1, 50	1.99	0.04	0.03
**DV: enjoyed lab low intensity session**							
Neuroticism	−0.25	[−0.42, −0.08]	0.005	1, 43	8.74	0.17	0.15
**DV: enjoyed easy long ride**							
Agreeableness	0.24	[0.01, 0.27]	0.049	1, 42	4.11	0.09	0.07
**DV: enjoyed threshold ride**							
Neuroticism	−0.19	[−0.35, −0.03]	0.024	1	1.29		
Openness	−0.25	[−0.40, −0.09	0.005	1	5.13		
Sex (male)	−0.63	[−1.21, −0.05]	0.031	1	4.73		
Regression			0.015	3, 51	3.85	0.18	0.14
**DV: enjoyed high intensity interval ride**							
Extraversion	0.21	[0.01, 0.43]	0.031	1	0.68		
Openness	−0.40	[−0.65, −0.15]	0.004	1	9.10		
Regression			0.012	1, 46	4.89	0.18	0.14
**DV: enjoyed lab VO**_2peak_ **test**							
Extraversion	0.13	[0.02, 0.25]	0.039	1, 117	4.32	0.04	0.03

The full models included all Big Five personality traits, age and sex.

There was an error in the **Results section**: Figure 4 was omitted. Figure 4 and its corresponding information are provided below.

Figure 4 should be cited in the following sentence, at the end of the **Results section**, under “*Intervention Outcomes*”.

Furthermore, participants who scored high on neuroticism reported a greater decrease in stress after the intervention, *F*_(1, 49)_ = 9.94, *p* = 0.003, $R_{\text{adj}}^{2}$ = 0.15 (Figure 4).

Figure 4 and its caption appear below.

**Figure 4:**
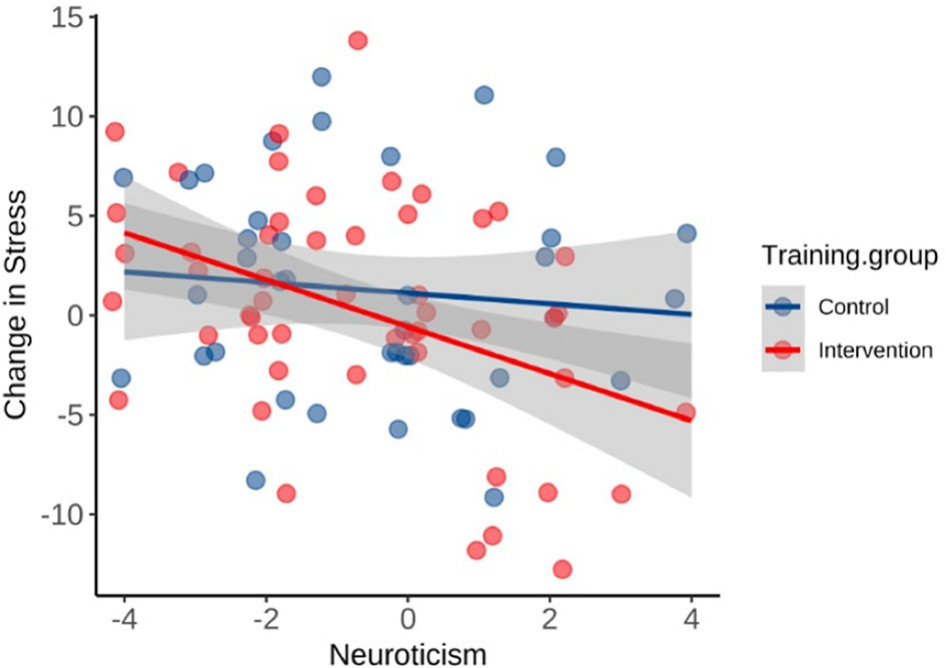
Relationship between neuroticism scores and changes in stress after the 8-week period. The prediction was significant in the intervention group only (*R*^2^ = 0.17, *p* = 0.003).

The original version of this article has been updated.

