# Supplementary material for: Personality traits can predict which exercise intensities we enjoy most, and the magnitude of stress reduction experienced following a training program
Source: Front Psychol. 2025 Jul 8;16:1587472. doi: 10.3389/fpsyg.2025.1587472 (PMC12279706; doi:10.3389/fpsyg.2025.1587472)
Supplement: Supplementary file 1 [file Supplementary_file_1.docx]

Supplementary Table 1 - Comparison of Pre and Post testing changes in physical variables for the Intervention and Control groups.

|  | Control | Intervention | p |
| --- | --- | --- | --- |
| Change in PA hours (1-5) | 0.0 ± 0.9 | 0.5 ± 0.9 * | .013 |
| Change in VO_2_max (ml/kg/min) | 0.3 ± 2.9 | 1.9 ± 3.4 * | .019 |
| Change in Load max (W) | 2 ± 17 | 12 ± 18 * | .013 |
| Change in RER max | 0.01 ± 0.08 | -0.01 ± 0.06 | .30 |
| Change in Press ups (n) | 2 ± 7 | 6 ± 8 * | .017 |
| Change in Plank time (s) | 3 ± 18 | 18 ± 23 *** | <.001 |
| Change in Weight (Kg) | -0.1 ± 1.7 | -0.2 ± 2.2 | .62 |

Supplementary Table 2 - Multiple linear regression outputs predicting fitness intervention outcomes from personality traits.

| Predictor | p | df | F | R^2^ | R^2^ _adj_ |
| --- | --- | --- | --- | --- | --- |
| DV: Change in peak cycling power output |  |  |  |  |  |
| Conscientiousness | .032 | 1, 49 | 4.89 | .09 | .07 |
| DV: Change in weekly exercise hours |  |  |  |  |  |
| Conscientiousness | .060 | 1, 46 | 3.70 | .07 | .05 |
| DV: Change RER max |  |  |  |  |  |
| Extraversion | .013 | 1, 48 | 6.70 | .12 | .10 |
| DV: Change in Stress |  |  |  |  |  |
| Neuroticism | .003 | 1, 49 | 9.95 | .17 | .15 |

Supplementary Table 3 – Logistic regression outputs to determine the likelihood of participants engaging in the intervention programme.

|  | df | OR | Wald | p |
| --- | --- | --- | --- | --- |
| DV: Recorded HR data |  |  |  |  |
| Neuroticism | 1 | 0.73 | -2.00 | .045 |
| DV: Returned for post testing |  |  |  |  |
| Extraversion | 1 | 0.70 | -2.27 | .023 |
| Openness | 1 | 1.42 | 1.96 | .048 |
| DV: Is part of an endurance club |  |  |  |  |
| Conscientiousness | 1 | 1.31 | 2.44 | .015 |
